# Consistent Estimation of Gibbs Energy Using Component Contributions

**DOI:** 10.1371/journal.pcbi.1003098

**Published:** 2013-07-11

**Authors:** Elad Noor, Hulda S. Haraldsdóttir, Ron Milo, Ronan M. T. Fleming

**Affiliations:** 1 Plant Sciences Department, Weizmann Institute of Science, Rehovot, Israel; 2 Center for Systems Biology, University of Iceland, Reykjavik, Iceland; 3 Luxembourg Centre for Systems Biomedicine, University of Luxembourg, Esch-sur-Alzette, Luxembourg; Medical College of Wisconsin, United States of America

## Abstract

Standard Gibbs energies of reactions are increasingly being used in metabolic modeling for applying thermodynamic constraints on reaction rates, metabolite concentrations and kinetic parameters. The increasing scope and diversity of metabolic models has led scientists to look for genome-scale solutions that can estimate the standard Gibbs energy of all the reactions in metabolism. Group contribution methods greatly increase coverage, albeit at the price of decreased precision. We present here a way to combine the estimations of group contribution with the more accurate reactant contributions by decomposing each reaction into two parts and applying one of the methods on each of them. This method gives priority to the reactant contributions over group contributions while guaranteeing that all estimations will be consistent, i.e. will not violate the first law of thermodynamics. We show that there is a significant increase in the accuracy of our estimations compared to standard group contribution. Specifically, our cross-validation results show an 80% reduction in the median absolute residual for reactions that can be derived by reactant contributions only. We provide the full framework and source code for deriving estimates of standard reaction Gibbs energy, as well as confidence intervals, and believe this will facilitate the wide use of thermodynamic data for a better understanding of metabolism.

## Author Summary

The metabolism of living organisms is a complex system with a large number of parameters and interactions. Nevertheless, it is governed by a strict set of rules that make it somewhat predictable and amenable to modeling. The laws of thermodynamics play a pivotal role by determining reaction feasibility and by governing the kinetics of enzymes. Here we introduce estimations for the standard Gibbs energy of reactions, with the best combination of accuracy and coverage to date. The estimations are derived using a new method which we denote *component contribution*. This method integrates multiple sources of information into a consistent framework that obeys the laws of thermodynamics, and provides a significant improvement in accuracy compared to previous genome-wide estimations of standard Gibbs energies. We apply and test our method on reconstructions of *E. coli* and human metabolism and, in addition, do our best to facilitate the use of these estimations in future models by providing open-source software that performs the integration in a streamlined process.

This is a *PLOS Computational Biology* Methods Article.

## Introduction

A living system, like any other physical system, obeys the laws of thermodynamics. In the context of metabolism, the laws of thermodynamics have been successfully applied in several modeling schemes to improve accuracy in predictions and eliminate infeasible functional states. For instance, several methodologies that reflect the constraints imposed by the second law of thermodynamics have been developed [1]–[3] and were shown to remove thermodynamically infeasible loops and improve overall predictions. Alternatively, thermodynamic data have been integrated directly into genome-wide models and analysis methods [4]–[10]. Unfortunately, this integration has been hindered by the fact that thermodynamic parameters for most reactions are effectively missing (sometimes due to scattered accessibility or non-standard annotations).

The nearly ubiquitous method for experimentally obtaining thermodynamic parameters for biochemical reactions, specifically their standard transformed Gibbs energies 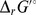, is directly measuring the apparent equilibrium constant 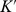 and then applying the formula 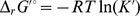, where 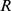 is the gas constant and 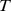 is the temperature. Typically, the substrates of the reaction are added to a buffered medium together with an enzyme that specifically catalyzes the reaction. After the concentrations reach a steady-state, the reaction quotient 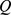 is calculated by dividing the product concentrations by the substrate concentrations. It is recommended to do the same measurement in the opposite direction as well (starting with what were earlier the products). If the experiment is successful, and the steady-state reached is an equilibrium state then both values for 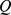 (measured in both directions) will be equal to 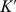 and thus to each other. Notably, due to the nature of this method, only reactions with 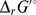 close to the equilibrium value of zero can be directly measured since current technology allows measuring metabolite concentrations only within a range of a few orders of magnitude. Although this method involves purifying substantial amounts of the enzyme, it has been applied to many of the enzyme-catalyzed reactions studied throughout the last century and the results were published in hundreds or even thousands of papers. A comprehensive collection of measured 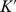 (among other thermodynamic parameters), for more than 400 reactions, has been published by the National Institute of Standards and Technology (NIST) in the Thermodynamics of Enzyme-Catalyzed Reactions Database (TECRDB [11]). However, even this wide collection covers less than 10% of biochemical reactions in standard metabolic reconstructions, such as the *E. coli* model iAF1260 [5].

In 1957 [12], K. Burton recognized that these apparent equilibrium constants can be used (together with chemically derived standard Gibbs energies for some simple compounds) to calculate equilibrium constants of reactions with no known 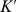 values. This method is based on the notion that by knowing the 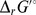 of two different reactions, one can calculate the 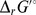 of the combined reaction by summing the two known standard transformed Gibbs energies – as dictated by the first law of thermodynamics. For example, although the reaction of ATP hydrolysis (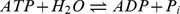) might be too far from equilibrium to be measured directly, one can more easily measure the 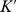 of the reactions of glucose kinase (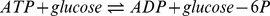; 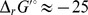 kJ/mol) and of glucose-6P phosphatase (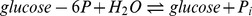; 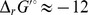 kJ/mol), which are both closer to equilibrium. The standard transformed Gibbs energy for the total reaction (i.e. ATP hydrolysis) would thus be 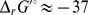 kJ/mol.

In order to facilitate these 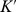 calculations, Burton published a table of about 100 inferred standard Gibbs energies of formation (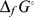) which are defined as the standard Gibbs energy 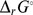 of the *formation reaction*, i.e. the reaction of forming a compound out of pure elements in their standard forms (e.g. 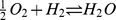). Some of these values were taken from chemical thermodynamic tables, and the rest were derived by Burton using the arithmetic approach of combining reactions. For instance, if all species except one in an enzyme-catalyzed reaction have known 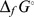, and the reaction's 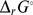 can be obtained experimentally, then the last remaining 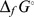 can be calculated and added to the table. After compiling such a table, the 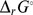 of any reaction involving species that appear in the table can be easily calculated by summing the formation energies of all the products and subtracting those of the substrates. Throughout this paper we will refer to this method of calculating 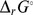 as the Reactant Contribution (RC) method, since it is based on the contribution of each reactant to 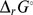 (i.e. its standard Gibbs energy of formation).

In the 50 years following Burton's work, several such tables of formation Gibbs energies have been published. Some of the most noteworthy are the table by R. Thauer [13] and the larger collection by R. Alberty [14], [15]. Using these values, one can determine Gibbs energies for more reactions at a wider range of physiological conditions (pH, ionic strength) than the set of reactions measured and stored in TECRDB. However, even this advanced method covers less than 10% of reactions in the *E. coli* model. This gap has prompted scientists to develop methods that can estimate the missing thermodynamic parameters for genome-wide models.

Quite coincidentally, a year after Burton published his thermodynamic tables, S. Benson and J. Buss [16] published their work on additivity rules for the estimation of molecular properties. Benson and Buss called the law of additivity of atomic properties a *zero-order* approximation, the additivity of bond properties a *first-order* approximation, and the additivity of group properties a *second-order* approximation. Groups were defined as pairs of atoms or structural elements with distances of 3–5 Å. The contribution of each group to the total was determined by linear regression. Using the second-order approximation, 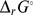 is calculated as the sum of the standard Gibbs energy contributions of groups that are produced in the reaction, minus the contributions of groups that are consumed. This method is commonly called the Group Contribution (GC) method. Burton's method of calculating the Gibbs energy of formation for compounds (which we denote RC) can be thought of as a 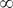-*order* approximation, where the entire molecule is taken as the basic additivity unit for estimating the 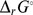 (of course, this is not actually an approximation).

**Figure 1:**
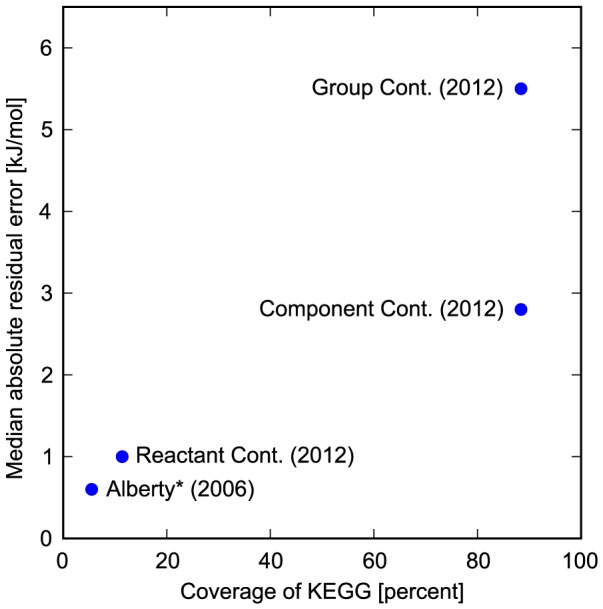
The development of Gibbs energy estimation frameworks. The coverage is calculated as the percent of the relevant reactions in the KEGG database (i.e. reactions that have full chemical descriptions and are chemically balanced). The median residual (in absolute values) is calculated using leave-one-out cross-validation over the set of reactions that are within the scope of each method. Note that the reason component contribution has a higher median absolute residual than RC is only due to its higher coverage of reactions (for reactions covered by RC, the component contribution method gives the exact same predictions). *The residual value for Alberty's method is not based on cross-validation since it is a result of manual curation of multiple data sources – a process that we cannot readily repeat.

Group contribution methods were relatively successful in estimating the thermodynamic parameters of ideal gases [16]–[19], and later extended to liquid and solid phases [20]. Only in 1988 [21] was it brought to the world of biochemical reactions in aqueous solutions and has since become the most widely used technique for estimating the Gibbs energy of reactions [22]–[24]. GC methods can cover the majority of relevant biochemical reactions (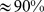 and 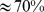 of the reactions in *E. coli* and human cell metabolic models respectively) [5], [10], [24]. The downside of GC lies in its accuracy, since it relies on a simplifying assumption that the contributions of groups are additive. Evidently, the average estimation error attributed to GC is about 9–10 kJ/mol [23]. In a recent study, we showed that an improvement of 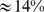 can be achieved by considering different pseudoisomers that exist simultaneously for many of the compounds [24] (see Section S1 in Text S1 for details). Even with this improvement, error in GC estimates is still significantly higher than in RC estimates (Figure 1).

In this paper, we aim to unify GC and RC into a more general framework we call the Component Contribution method. We demonstrate that component contribution combines the accuracy of RC with the coverage of GC in a fully consistent manner. A plot comparing the component contribution method to other known methods is given in Figure 1.

### Unifying reactant and group contribution methods

The extensive use of formation Gibbs energies for calculating 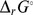 might create the impression that combining these two frameworks (RC and GC) is a trivial task. Traditionally, the formation energy of all pure elements in their standard forms is set to zero by definition. All other compounds' formation energies are calculated in relation to these reference points. This is a sound definition which creates a consistent framework for deriving the 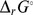 of any reaction which is chemically balanced. However, the difficulty of calculating the formation energy for some complex but useful co-factors has been side-stepped by creating a somewhat looser definition of formation Gibbs energy, where several non-elemental compounds are defined as reference points as well (with a standard formation energy of zero). For some rare reactions, this definition can create a conflict that will result in a very large mistake in the 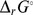.

For instance, Alberty's formation energy table [15] lists 16 compounds as having 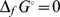. Among these, only 5 are elements (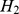, 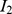, 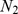, 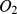, and 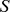) and 11 are co-factors (CoA^−1^, NAD(ox)^−1^, FAD(ox)^−2^, FMN(ox)^−2^ and seven other redox carriers). In most reactions which use these co-factors as substrates, the “zeros” will cancel out since one of the products will match it with a formation energy which is defined according to the same reference point (e.g. FAD(ox)^−2^ will be matched with FAD(red)^−2^ whose formation energy is 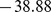 kJ/mol in Albery's table). Nevertheless, there are a handful of reactions where this matching doesn't occur. In the reaction 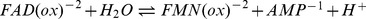 (catalyzed by *FAD nucleotidohydrolase*, EC 3.6.1.18), there is a violation of conservation laws for FAD and FMN (both have 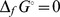 in Alberty's table). Therefore, using the table naïvely for this reaction would yield a wrong value, namely 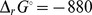 kJ/mol. Combining formation energies derived using GC with ones from RC greatly increases the number of reactions where different reference-points are mixed together, and mistakes such as the one described above become much more common.

**Figure 2:**
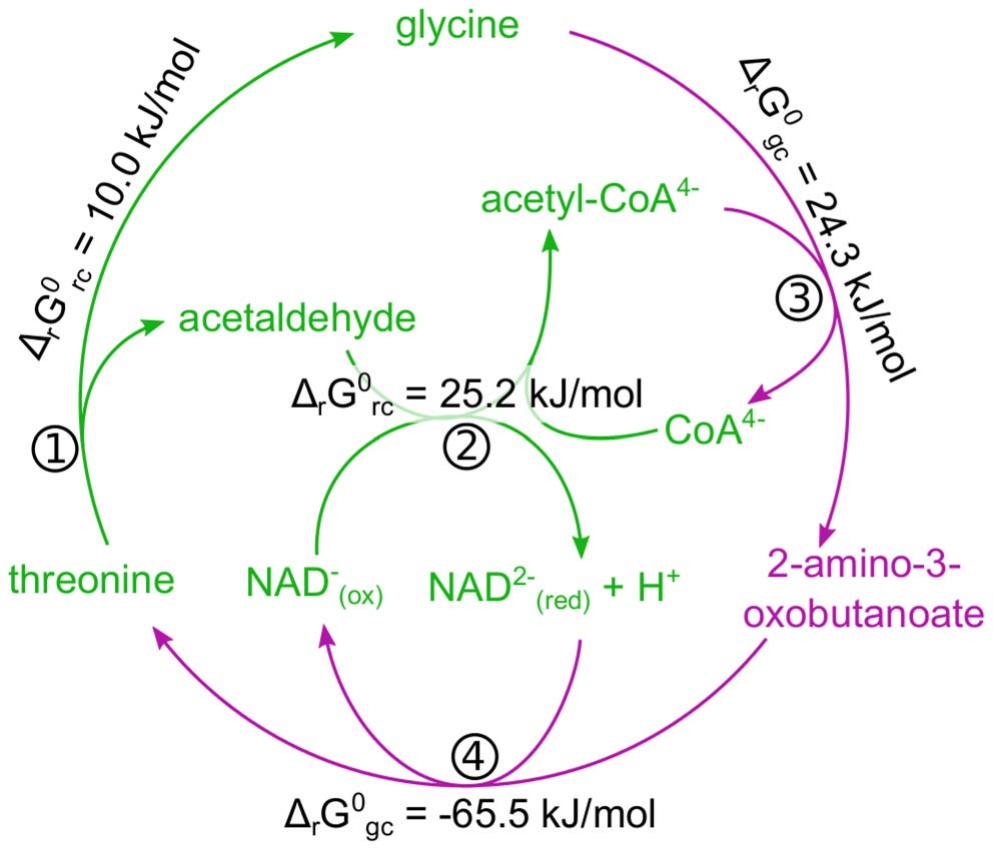
An example of a futile cycle where Gibbs energies are derived using RC and GC. The combined stoichiometry of (1) threonine aldolase, (2) acetaldehyde dehydrogenase (acetylating), (3) glycine C-acetyltransferase, and (4) threonine:NAD oxidoreductase creates a futile cycle where all the inputs and outputs are balanced. Using RC we are able to derive the 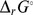 of reactions 1 and 2 (green), but since 2-amino-3-oxobutanoate does not appear in formation energy tables – we must use GC for reactions 3 and 4 (magenta). The sum of all 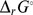 in this case is −6.0 kJ/mol which is a violation of the first law of thermodynamics.

One way to deal with the problem of reference-point conflicts, is to use either RC or GC exclusively for every reaction 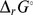 being estimated. Specifically, RC will by applied to all reactions that can be completely covered by it. Only if one or more reactants are missing from the formation energy table, we would use the less precise GC method for the entire reaction. Unfortunately, combining the frameworks in such a way can easily lead to violations of the first law of thermodynamics. This stems from the fact that inconsistent use of formation energies across several reactions, coming from inaccuracies in the estimation method that do not cancel out, can create situations where futile cycles will have a non-zero change in Gibbs energy. An example for such a futile cycle is given in Figure 2. Applying this method for large-scale metabolic reconstructions will most likely lead to non-physical solutions.

Reference-point conflicts and first-law violations can both be avoided, by adjusting baseline formation energies of compounds with non-elemental reference points to match group contribution estimates. This approach was taken in [8] and [10]. The formation energies of FAD(ox)^−2^ and all other reference points in Alberty's table were set equal to their group contribution estimates. All formation energies that were determined relative to each reference point were then adjusted according to Alberty's table to maintain the same relative formation energies. The main disadvantage of this approach is that the set of reference points is fixed and limited to a few common cofactors. The coverage of reactant contributions could be increased by also defining less common metabolites as reference points, but listing them all in a static table would be impractical and inefficient.

The component contribution method, which is described in detail in the following sections of this paper, manages to combine the estimates of RC and GC while avoiding any reference-point conflicts or first-law violations. In the component contribution framework, the maximal set of reference points given a set of measured reactions is automatically determined. We maintain the notion of prioritizing RC over GC, but rather than applying only one method exclusively per reaction, we split every reaction into two independent reactions. One of these sub-reactions can be evaluated using RC, while the other cannot – and thus its 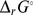 is calculated using GC. We use linear orthogonal projections in order to split each of the reactions, ensuring that all estimated 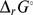 values are self-consistent. The choice of orthogonal projections is somewhat arbitrary, and is based on the assumption that it is beneficial to minimize the euclidean distance to the projected point that can be estimated using RC. This framework also enables us to calculate confidence intervals for standard reaction Gibbs energies in a mathematically sound way.

## Results

### The component contribution method

The component contribution method integrates reactant contributions and group contributions in a single, unified framework using a layered linear regression technique. This technique enables maximum usage of the more accurate reactant contributions, and fills in missing information using group contributions in a fully consistent manner. The inputs to the component contribution method are the stoichiometric matrix of measured reactions, denoted 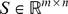, and a list of measurements of their standard Gibbs energies 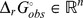 (see Table S2 in Text S1 for a list of mathematical symbols). In our case, all data is taken from TECRDB [11] and tables of compound formation energies [13], [15]. As a pre-processing step which is used to linearize the problem, we apply an inverse Legendre transform to the observed equilibrium constants in TECRDB and the formation energies, if necessary (same as in [24], see Section S1 in Text S1). To provide context for the mathematical formulation of the component contribution method, we precede it with general formulations of the reactant and group contribution methods, and discuss the limitations of each. The reactant and group contribution methods are both based on linear regression. The difference between the two methods lies in the regression models used in each.

#### Reactant contribution method

The regression model used in the reactant contribution method is based on the first law of thermodynamics (conservation of energy). The first law dictates that the overall standard Gibbs energy of a reaction that takes place in more than one step, is the sum of the standard Gibbs energies of all the intermediate steps at the same conditions [25]. Consequently, if 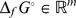 is the vector of standard Gibbs energies of formation for compounds in 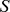, then the standard Gibbs energies of reactions in 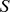 are given by the equation
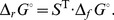
(1)


From Eq. 1 it is apparent that 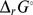 is in the range of 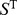, which we denote by 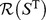. In practice, this may not be readily true for 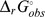 from TECRDB, since its values are empirically derived and thus subject to measurement noise. Also, the exact ionic strength is not known for most measurements and the extended Debye-Hückel theory of electrolyte solutions [26] (which the inverse Legendre transform is based on [27]) is itself an approximation. The linear regression model used in the reactant contribution method for 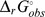 therefore takes the form
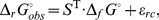
(2)where 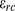 encompasses the error from the aforementioned sources.

Least-squares linear regression on the system in Eq. 2 gives the reactant contribution estimate of the standard Gibbs energies of formation for compounds in 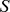


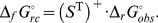
(3)The Moore–Penrose pseudoinverse 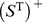 is used because 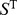 is typically column rank deficient. Reactant contribution fitted standard Gibbs energies for reactions in 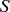 are,
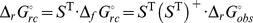
(4)i.e., they are the orthogonal projection of 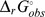 onto 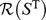. 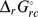 is therefore the closest point to 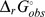 that is consistent with the first law of thermodynamics. The residual of the fit
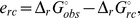
(5)gives an estimate of the error term 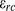 in Eq. 2. We stress that there is a conceptual distinction between the residual (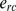) and the statistical error (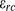). 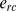 is dependent on the specific sample of equilibrium constants we use in the training set, while 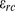 is a random variable that can only be approximated. We use the term *error* for the deviation of an observed or estimated Gibbs energy (known values), from the (unknown) true Gibbs energy. The term *residual* is used for the deviation of an observed Gibbs energy from an estimate. We note that 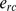 is in the null space of 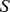, denoted 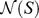, since the null space is the orthogonal complement of 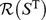, according to the fundamental theorem of linear algebra.

The standard Gibbs energy 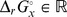 of an unmeasured reaction with stoichiometric vector 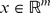 can be estimated with the reactant contribution method as
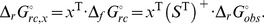
(6)This result is consistent with the first law of thermodynamics in the following sense. In general, the first law implies that the standard Gibbs energy of a linear combination of reactions, is the same combination applied to the respective standard reaction Gibbs energies. Mathematically, if 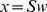 then 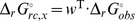. The former equation gives 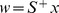 which is precisely the result in Eq. 6. Having compliance with the first law as the only assumption explains the high accuracy of the reactant contribution method.

The reactant contribution method can be used to evaluate standard Gibbs energies for 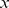 in the range of 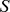, i.e. reactions that are linear combinations of reactions in 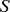 (and thus have at least one solution for 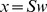). Any component of 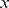 that is not in 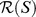 cannot be evaluated. Since 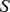 is rank deficient, its range represents only a fraction of the entire space of reactions and thus most reactions are under-determined by this method. For instance, the CMP phosphohydrolase reaction (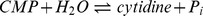) exists in the *E. coli* model but is not listed as a measured reaction in TECRDB. Although CMP and cytidine both appear in other measured reactions (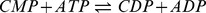 and 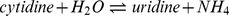), it is impossible to use a combination of reactions in TECRDB to find the 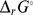 of the CMP phosphohydrolase reaction.

#### Group contribution method

Increased reaction coverage can be achieved using the group contribution method, where each compound in 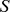 is decomposed into a predefined set of structural subgroups. Each decomposition is represented by a row of the group incidence matrix 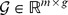, and 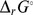 is assumed to be a linear combination of the standard Gibbs energy contributions 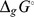 of the groups in 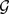. The linear regression model for the group contribution method is
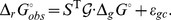
(7)

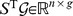
 describes the group decompositions of reactions in 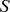 i.e., the stoichiometry of groups that are consumed or produced in the reactions. An estimate of 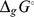 is obtained using linear regression on the system in Eq. 7 i.e.,
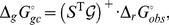
(8)and like in reactant contribution we define 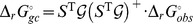 and 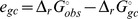. The group contribution estimate of the standard reaction Gibbs energy for 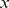 can then be derived as
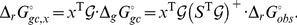
(9)


The reaction coverage of the group contribution method is much greater than that of the reactant contribution method in Eq. 6, because the column rank deficiency of 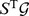 is much smaller than that of 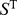. However, this greater coverage comes at a price, since the assumption of group additivity underlying the model in Eq. 7 is not always accurate. We estimated the root-mean-square error resulting from this assumption as 6.8 kJ/mol for all reactions in 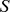 (see Section S4 in Text S1 for details). The reaction coverage of group contribution methods is still limited to 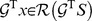, i.e. reactions with group decompositions that are linear combinations of the group decompositions of measured reactions.

#### Mathematical formulation of the component contribution method

**Figure 3:**
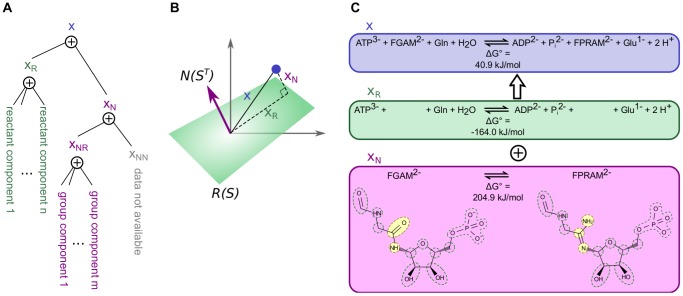
A diagram illustrating how the component contribution method projects the stoichiometric vector onto the different spaces. (A) The reaction vector 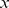 is decomposed into the two components 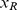 and 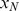, where the reactant contribution and group contribution methods are used for the relevant components. Later, 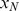 is decomposed into 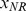 and 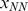. The same projection is shown graphically in (B) where the green plane represents the range of 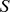 and the normal to that plane represents the null space of 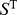. (C) An example for a reaction which decomposes into two non-zero components. In this case, the component 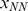 is equal to 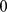, which means that the reaction is covered by the component contribution method.

The reactant contribution method covers any vector in the range of 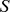. The component contribution method takes advantage of the fact that any reaction vector 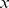 in 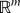 can be decomposed into a component 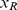 in the range of 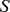, and an orthogonal component 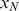 in the null space of 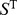. Let 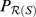, 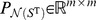 be the orthogonal projection matrices onto the range of 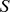 and the null space of 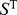, respectively. Then 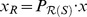 and 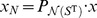 (so 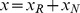 and 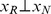). The component contribution method applies the more reliable reactant contribution method to evaluate 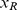, and only applies the less reliable group contribution method to 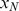 (see Figure 3). The standard reaction Gibbs energy estimate for 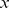 is obtained by summing up the contributions from the two components (see Equations 6 and 9) i.e.,
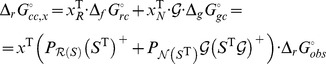
(10)(see the full derivation in Section S3 in Text S1). We note that using the two orthogonal projections is only one option for separating 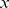 to two components and applying RC and GC on each one respectively. Other pairs of linear projections could be applied as long as they fulfill the requirement that they sum up to the identity matrix, and that the range of the first one is 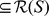. Here we chose 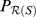 and 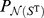 because they minimize the norm of the second component, and we assume there is benefit to it.

The component 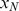 in the null space of 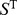 can only be evaluated if 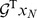 is in the range of 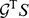, i.e. the space covered by group contributions. We thus require that 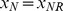 where 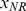 is the component of 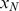 in 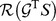. If 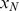 has a nonzero component 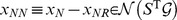 then the overall reaction 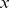 cannot be evaluated using component contributions. In practice we assign an infinite uncertainty to reactions where 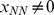 as discussed in section *Calculation of confidence intervals*. The two orthogonal components of 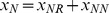 are determined by orthogonal projections onto the subspaces of 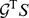, in the same way that 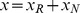 was decomposed by projections onto the subspaces of 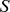. Component contribution is thus based on two layers of orthogonal decompositions; a first layer where 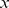 is decomposed into 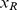 and 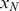, and a second layer where 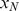 is decomposed into 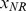 and 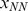 (Figure 3).

A common example where 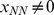 occurs where 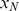 is a reaction that includes the formation of an uncommon group. If this group does not appear (or is always conserved) in all of the reactions in the training set then the contribution of that group is unknown. Since 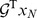 has a non-zero value corresponding to that group, 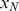 cannot be in the range of 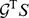.

### Validation results

**Figure 4:**
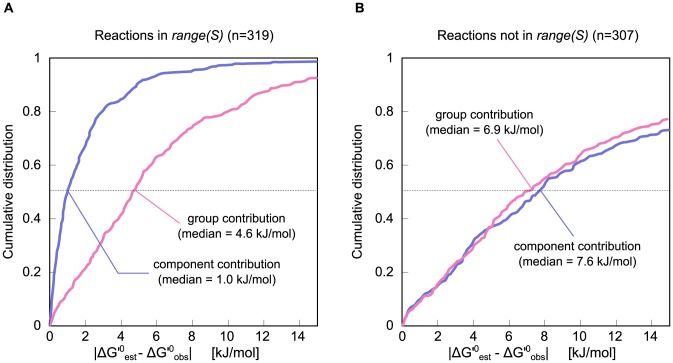
Cumulative distributions for the cross-validation results. The CDF of the absolute-value residuals for both group contribution (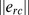, pink) and component contribution (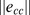, purple). The reactions were separated to ones which are (A) linearly-dependent on the set of all other reactions (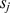 is in the range of 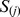, the stoichiometric matrix of all reactions except 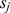), and (B) to ones which are linearly-independent (and thus component contribution uses group decompositions for at least part of the reaction). We found an 80% reduction in the median for the former set and no significant change for the latter (p-value = 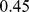).

In order to evaluate the improvement in estimations derived using component contribution compared to an implementation of group contribution [24], we ran a cross-validation analysis (see section *Leave-one-out cross-validation* for details). The results of this analysis are shown in Figure 4, where we compare the distributions of the absolute residuals (the difference between each method's estimated 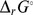 and the observed 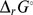 according to TECRDB). For each estimation, the value of 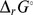 for that reaction (or any other measurement of the same reaction) was not used for training the group contribution and component contribution methods.

Our results show a significant improvement for component contribution compared to group contribution when focusing on reactions in the range of 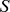. The median of all residuals (absolute value) was reduced from 4.6 to 1.0 kJ/mol (p-value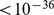) for this set of reactions. For reactions that were not in 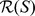, there was no significant difference (p-value = 0.45) in the median absolute residual between the two methods. The error in group contribution estimates that is due to the assumption of group additivity does not depend on the extent to which group contribution is used (see Section S4.2 in Text S1). Because component contribution uses group contribution to some extent for all reactions that are not in 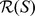, the error in component contribution estimates for those reactions is not significantly lower than the error in group contribution estimates. Note that it is still very important to use component contribution for these reactions (and not GC) for the sake of having consistent estimations across whole metabolic models (see section *Unifying reactant and group contribution methods* in the Introduction).

In each iteration of the cross-validation, one reaction was excluded from the training set. To further validate the component contribution method, we used the results of each iteration to predict independent observations of the reaction that was excluded. All available observations of that reaction were then compared against the prediction intervals for its standard Gibbs energy (see section *Calculation of prediction intervals* in the Methods). Overall, we found that 73% of observations fell within their respective 68% prediction intervals, 89% fell within their 90% prediction intervals, 92% fell within their 95% prediction intervals, and 97% within their 99% intervals. Prediction intervals obtained with the component contribution method were on average 36% smaller than those obtained with group contribution. Taken together, these results show that the component contribution method yields estimates with reliable confidence intervals, as well as increased accuracy and reduced uncertainty compared to group contribution.

### Application to genome-scale metabolic reconstructions

A major application of the component contribution method is estimation of standard Gibbs energies for reactions in genome-scale reconstructions. Such large reaction networks require consistent and reliable estimates with high coverage. If estimates are not consistent, the risk of reference point violations increases with network size. As discussed in section *Adjustment to in vivo conditions*, metabolic models generally require estimates of standard transformed Gibbs energies, 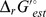, at *in vivo* conditions. To meet this requirement, we have integrated the component contribution method into a new version (2.0) of von Bertalanffy [28] (see section *Implementation and availability of code*).

Here, we apply von Bertalanffy 2.0 to two reconstruction; the *E. coli* reconstruction iAF1260 [5] and the human reconstruction Recon 1 [29]. Standard transformed reaction Gibbs energies had previously been estimated for both reconstructions, with older versions of von Bertalanffy [8], . Those older versions relied on a combination of experimentally derived standard formation energies from [15], and estimated standard formation energies obtained with the group contribution method presented in [23]. We compare estimates obtained with the new version of von Bertalanffy, to both experimental data in TECRDB, and estimates obtained with the older versions.


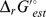
 were obtained for 90% (1878/2078) of internal reactions in iAF1260, and 72% (2416/3362) of internal reactions in Recon 1. External reactions i.e., exchange, demand and sink reactions are not mass or charge balanced and therefore have no defined Gibbs energies. To validate our estimates we compared them to available experimental data. Measurements of apparent equilibrium constants (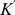) were available in TECRDB for 163 of the evaluated iAF1260 reactions, and 186 Recon 1 reactions. Multiple measurements, made at different experimental conditions, were often available for a single reaction. To enable comparison, the data in TECRDB was first normalized to standard conditions by applying an inverse Legendre transform as described in Section S1 in Text S1. The resulting standard reaction Gibbs energies (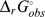) were then adjusted to the conditions in Tables 1 and 2 with von Bertalanffy, to obtain standard transformed reaction Gibbs energies, 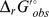. Comparison of 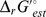 to 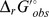 gave a root mean square error (RMSE) of 2.7 kJ/mol for iAF1260, and 3.1 kJ/mol for Recon 1.

**Table 1:** pH and electrical potential in each compartment of the *E. coli* reconstruction iAF1260.

Compartment	pH	Electrical potential (mV)
Cytosol	7.70	0
Periplasm	7.70	90
Extracellular fluid	7.70	90

Electrical potential in each compartment is relative to electrical potential in the cytosol. Temperature was set to 310.15 K (37°C), and ionic strength was assumed to be 0.25 M [14] in all compartments. Taken from [8].

**Table 2:** pH and electrical potential in each compartment of the human reconstruction Recon 1.

Compartment	pH	Electrical potential (mV)
Cytosol	7.20	0
Extracellular fluid	7.40	30
Golgi apparatus	6.35	0
Lysosomes	5.50	19
Mitochondria	8.00	−155
Nucleus	7.20	0
Endoplasmic reticulum	7.20	0
Peroxisomes	7.00	12

Electrical potential in each compartment is relative to electrical potential in the cytosol. Temperature was set to 310.15 K (37°C), and ionic strength was assumed to be 0.15 M [14] in all compartments. Taken from [10].

von Bertalanffy 2.0 relies on component contribution estimated standard reaction Gibbs energies, whereas older versions relied on a combination of experimental data and group contribution estimates. Table 3 compares standard transformed Gibbs energy estimates, for iAF1260 and Recon 1, between versions. Use of component contribution resulted in both higher coverage and lower RMSE than was achieved with the previously available data. The greater coverage was due to reactions where groups or compounds that were not covered by component contributions canceled out, because they appeared unchanged on both sides of the reactions. Such reactions are easily identified and evaluated within the component contribution framework.

**Table 3:** Comparison of standard transformed reaction Gibbs energy estimates based on component contributions, to estimates based on previously available data.

	iAF1260		Recon 1	
	Fleming et al. [8]	Current study	Haraldsdóttir et al. [10]	Current study
Coverage	85%	90%	63%	72%
RMSE (kJ/mol)	9.9	2.7	11.6	3.1
Mean 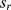 (kJ/mol)	20.3	2.3	3.4	2.2

Another improvement achieved with the component contribution method was the lower standard error, 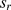, of standard reaction Gibbs energy estimates compared with previously available methods (Table 3). This is an important improvement as standard error has previously been shown to affect predictions made based on reaction Gibbs energy estimates [6], [8], [10]. The reduction in 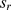 was obtained by accounting for covariances in parameter estimates (see section *Calculation of confidence intervals*). As we showed in section *Validation results*, the lower standard errors of component contribution estimates yielded reliable prediction intervals for observed standard reaction Gibbs energies. They can therefore be expected to also yield reliable confidence intervals for true standard reaction Gibbs energies.

**Figure 5:**
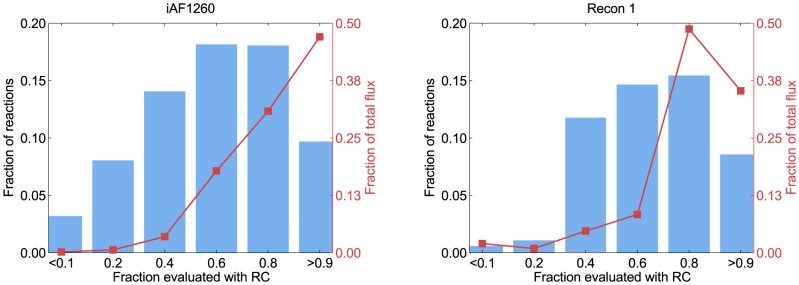
Distribution of the fractions of reaction vectors (black) in iAF1260 (*E. coli*) and Recon 1 (human), that were in the range of 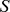, and were thus evaluated with reactant contribution (RC). For a reaction 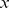, this fraction was calculated as 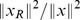. Passive and facilitated diffusion reactions, where the reactants undergo no chemical changes, are not included in the figure. 9.4% of all evaluated reactions in iAF1260 were fully evaluated using only reactant contributions. These reactions carried approximately half of the total flux (red) in 312 predicted flux distributions. The 8.3% of evaluated reactions in Recon 1 that were fully evaluated with reactant contributions, carried close to a third of the total flux in 97 predicted flux distributions.

The lower RMSE achieved with component contribution stems primarily from two factors. The first is the normalization of the training data by the inverse Legendre transform, which in [24] was shown to lead to significant improvements in group contribution estimates of Gibbs energies. The second factor is the greater number of reactions that are fully evaluated with reactant contribution (Eq. 6). Close to 10% of all evaluated reactions in both iAF1260 and Recon 1, were fully evaluated using only reactant contribution (Figure 5). Although this category represents a minority of all reactions, it includes the majority of reactions in central carbon metabolism. The greater accuracy in Gibbs energy estimates for reactions in central carbon metabolism is expected to have a disproportionally large effect, as these reactions are involved in most metabolic activities. To support this claim, we predicted 312 flux distributions for iAF1260 and 97 flux distributions for Recon 1 (see Section S6 in Text S1 for details). We found that the tenth of reactions that were fully evaluated with reactant contributions carried approximately half of the total flux in iAF1260 and a third of the total flux in Recon 1 (Figure 5).

## Discussion

The component contribution method presented in this paper merges two established methods for calculating standard Gibbs energies of reactions while maintaining each of their advantages; accuracy in the case of reactant contribution (RC) and the wide coverage of group contribution (GC). By representing every reaction as a sum of two complementary component reactions, one in the subspace that is completely covered by RC and the other in the complementary space, we maximize the usage of information that can be obtained with the more accurate RC method. Overall, we find that there is a 50% reduction in the median absolute residual compared to standard GC methods, while providing the same wide coverage and ensuring that there are no reference-point inconsistencies that otherwise lead to large errors. Furthermore, since our method is based on least-squares linear regression, we use standard practices for calculating confidence intervals for standard Gibbs energies (see section *Calculation of confidence intervals*), and for weighing the measured standard Gibbs energies used as training data (see Section S1.2 in Text S1).

Since the empirical data used in our method is measured in various conditions (temperature, pH, ionic strength, metal ion concentrations, etc.) – it is important to “standardize” the input data before applying any linear regression model [24]. In this work, we used an inverse Legendre transform to normalize the pH and ionic strength, but ignore the temperature effect and the metal ion concentrations (see Section S1.1 in Text S1). In addition, the proton dissociation constants were obtained from a third party software estimator (by Marvin, see Methods) and have a mean absolute error of about 0.9 pH units [30]. Notably, a commendable effort for creating a database of thermodynamic quantities [31] has been published recently, where the data was standardized using more reliable parameters and considering more effects. This database currently only covers reactions from glycolysis, the tricarboxylic acid cycle, and the pentose phosphate pathway. Therefore, we chose to use the more extensive TECRDB database and perform the inverse Legendre transform ourselves, effectively increasing the coverage while compromising on the accuracy of the data. Since the changes brought forward in the component contribution method are independent of the source of input data, we believe that it will benefit from any future improvements in these databases.

The precision of the component contribution method is limited by the accuracy of the measured reaction equilibrium constants used in the regression model. In cases of isolated reactions, where the empirical data cannot be corroborated by overlapping measurements, large errors will be directly propagated to our estimate of those reactions' standard Gibbs energies. As the number of measurements underlying an estimate is reflected in its standard error, however, confidence intervals for such reactions will be large. It is therefore recommended to use confidence intervals, and not point estimates, for simulations and predictions based on standard Gibbs energy estimates. In the future, it might be worthwhile to integrate several promising computational prediction approaches [32] which are not based on RC and GC, such as molecular mechanics methods [33], density functional methods [34], and post Hartree-Fock approaches [35], [36]. Although the computational cost of these methods can be substantial depending on the theoretical method and the solvation models [37] used, they have the advantage of being based on computable chemical and physical principles, implying that a 100% coverage of all biochemical reactions is achievable (though not yet practical). Currently, the accuracy of these methods for reactions in solution is limited. Nevertheless, they might already be useful for estimating 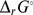 of reactions that are not covered by component contributions, or for validating the sparse measurements. Alternatively, a method that infers 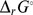 from reaction similarities named IGERS [38] manages to be much more accurate than GC when predicting the standard Gibbs energy of reactions which are very similar to a reaction with a measured 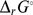. Adding IGERS as another layer between RC and GC using the ideas presented in this paper might contribute to the overall accuracy of our estimations. Finally, the laws of additivity suggested by [16] include single atom (zero-order) and single bond (first-order) contributions, which would be too crude to use for approximating Gibbs energies directly, but might be useful as two extra layers in a method like component contribution and help cover a wider fraction of the reaction space.

The use of thermodynamic parameters in modeling living systems has been hindered by the fact that it is mostly inaccessible or requires a high level of expertise to use correctly, especially in genome-scale models. In order to alleviate this limitation, we created a framework that facilitates the integration of standard reaction Gibbs energies into existing models and also embedded our code into the openCOBRA toolbox. The entire framework (including the source code and training data) is freely available. We envisage a collaborative community effort that will result in a simple and streamlined process where these important thermodynamic data are widely used and where future improvements in estimation methods will seamlessly propagate to modelers.

## Methods

### Calculation of confidence intervals

The component contribution estimated standard Gibbs energy 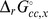 in Eq. 10, is a point estimate of the true standard Gibbs energy 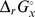 for reaction vector 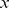. To quantify the uncertainty in this estimate, we need to calculate confidence intervals for 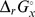. An important advantage of integrating the reactant and group contribution methods in a single, unified framework is that it greatly simplifies calculation of confidence intervals. We present the key equations in this section. A summary of the statistical theory underlying these equations [39] is given in Section S7 in Text S1.

The covariance matrix 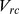 for the reactant contribution estimates (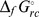 in Eq. 3) is calculated as
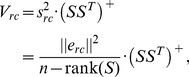
(11)where the matrix 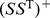 is scaled by the estimated variance 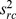 of the error term 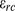 in Eq. 2. Our estimate of the variance was 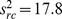 (kJ/mol)^2^. The covariance matrix 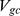 for the group contribution estimates (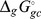) is likewise obtained as
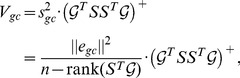
(12)where the estimated variance of 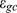 from Eq. 7 was 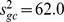 (kJ/mol)^2^.

For a reaction 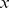, the *standard error* of 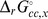 is given by
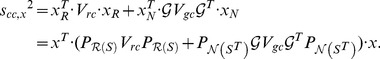
(13)The confidence interval for 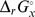, at a specified confidence level 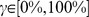, is given by
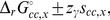
(14)where 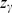 is the value of the standard normal distribution at a cumulative probability of 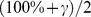. The 95% confidence interval for 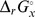 is therefore 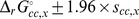.

In calculating 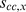, we employ the covariance matrices for estimated parameters 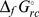 and 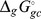. In contrast, Jankowski et al. used only the diagonal of the covariance matrix for 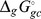 in their implementation of the group contribution method [23]. The main advantage of using covariance matrices is that it leads to more appropriate confidence intervals for 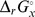, that can be much smaller. Knowledge about the relative Gibbs energy of two groups or compounds, increases with the number of measurements for reactions where those groups or compounds occur together. This knowledge should be reflected in smaller confidence intervals for reactions where the groups or compounds co-occur. Covariance matrices provide a means for propagating this knowledge. If only the diagonal of the covariance matrix is used, this knowledge is lost and confidence intervals will often be unnecessarily large.

The covariance matrices can likewise be used to propagate lack of knowledge to 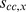. If 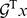 is not in 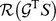 then the reaction 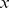 is not covered by the group contribution method or by the component contribution method. Then 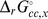 obtained with Eq. 10 will not be a valid estimate of 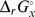, and should have a large (infinite) standard error. This can be achieved by adding a term to Eq. 13;
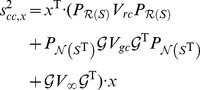
(15)where 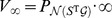, and 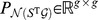 is a projection matrix onto the null-space of 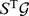. Eq. 15 will give 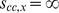 for all reactions that cannot be evaluated with component contributions because 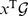 has a nonzero component in the null-space of 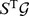. In practice, we use a very large value instead of 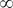 (e.g. 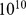 kJ/mol) which will dominate any reasonable Gibbs energy in case 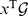 is not orthogonal to this null-space.

### Leave-one-out cross-validation

Both group contribution and component contribution are parametric methods that use a set of training data in order to evaluate a long list of parameters. In order to validate these models, we need to use more empirical data which has not been used in the training phase. Since data regarding reaction Gibbs energies is scarce, we apply the leave-one-out method in order to maximize the amount of data left for training in each cross-validation iteration. As a measure for the quality of the standard Gibbs energy estimations from each method we use the median absolute residual of the cross-validation results compared to the observations.

Our entire training set consists of 4146 distinct reaction measurements. However, since many of them are experimental replicates – measurements of the same chemical reaction in different conditions or by different researchers – we can only use each distinct reaction once. We thus take the median 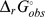 over all replicates (after applying the inverse Legendre transform) as the value to be used for training or cross-validation. We choose the median rather than the mean to avoid sensitivity to outliers. After this process of unifying observations, we are left with 694 unique reaction observations. Note that the repetitions do play a role in determining the standard error in standard Gibbs energy estimates (see section *Calculation of confidence intervals*). Finally, the vector of 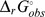 values for the unique reactions is projected onto the range of 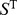 since we assume that the actual values comply with the first law of thermodynamics (see section *Reactant contribution method*) and that any deviation is caused by experimental error.

### Calculation of prediction intervals

The 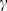 prediction interval for a reaction 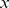, with estimated standard Gibbs energy 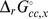, is calculated as
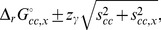
(16)where 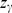 was defined in Eq. 14, and 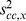, the standard error of 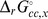, was defined in Eq. 15. 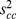 is calculated as
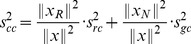
(17)i.e., it is a weighted mean of the estimated variances for reactant and group contribution, where the weights are the fractions of 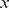 that are in 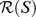 and 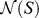, respectively. A summary of the statistical theory underlying calculation of prediction intervals [39] is given in Section S7 in Text S1.

### Adjustment to *in vivo* conditions

For an input reaction 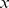, the component contribution method outputs an estimate of the reaction's standard *chemical* Gibbs energy 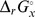. In a chemical reaction each compound is represented in a specific protonation state. This is in contrast to biochemical reactions, where each compound is represented as a pseudoisomer group of one *or more* species in different protonation states. To thermodynamically constrain models of living organisms we require Gibbs energies of biochemical reactions at *in vivo* conditions, known as standard *transformed* reaction Gibbs energies 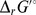.

We estimated 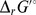 with version 2.0 of von Bertalanffy [8], [10], [28]; a Matlab implementation of biochemical thermodynamics theory as presented in [14]. A comprehensive summary of the relevant theory is given in [10]. In addition to component contribution estimates of standard Gibbs energies, required inputs to von Bertalanffy are a stoichiometric matrix 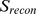 for a metabolic reconstruction of an organism, 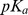 values for compounds in 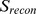, and literature data on temperature, pH, ionic strength (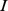) and electrical potential (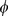) in each cell compartment in the reconstruction.

We estimated 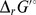 for reactions in two multi-compartmental, genome scale metabolic reconstructions; an *E. coli* reconstruction iAF1260 [5], and a human reconstruction Recon 1 [29]. The environmental parameters pH, 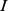 and 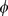 were taken from [8] for *E. coli* (Table 1), and from [10] for human (Table 2). 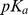 values were estimated with Calculator Plugins, Marvin 5.10.1, 2012, ChemAxon (http://www.chemaxon.com).

### Implementation and availability of code

The component contribution method has been implemented in both Matlab and Python. The Matlab implementation is tailored towards application to genome-scale metabolic reconstructions. It is fully compatible with the COBRA toolbox [40] and is freely available as part of the openCOBRA project on Sourceforge (http://sourceforge.net/projects/opencobra/). The component contribution method has been integrated into version 2.0 of von Bertalanffy to provide an easy-to-use tool to estimate transformed Gibbs energies at *in vivo* conditions. The Python implementation is a stand-alone package that can be used by researchers with suitable programming skills. The Python package includes a simple front-end called eQuilibrator (http://equilibrator.weizmann.ac.il/), which is a freely available online service. The Python code for component contribution is licensed under the open source MIT License and available on GitHub (https://github.com/eladnoor/component-contribution). Our code depends on the open source chemistry toolbox called Open Babel [41].

## Supporting Information

Text S1 Supporting text with sections on 1) the inverse Legendre transform of the training data, 2) group decomposition, 3) the full mathematical derivation of the component contribution method, 4) estimation of error in the group model, 5) reaction type statistics, 6) prediction of flux distributions, 7) the theory underlying calculation of confidence and prediction intervals, and 8) mathematical symbols used throughout the manuscript.
(PDF)
