# Supplementary material for: Consistent Estimation of Gibbs Energy Using Component Contributions
Source: PLoS Comput Biol. 2013 Jul 11;9(7):e1003098. doi: 10.1371/journal.pcbi.1003098 (PMC3708888; doi:10.1371/journal.pcbi.1003098)
Supplement: Text S1 — Supporting text with sections on 1) the inverse Legendre transform of the training data, 2) group decomposition, 3) the full mathematical derivation of the component contribution method, 4) estimation of error in the group model, 5) reaction type statistics, 6) prediction of flux distributions, 7) the theory underlying calculation of confidence and prediction intervals, and 8) mathematical symbols used throughout the manuscript. (PDF) [file pcbi.1003098.s001.pdf]

Text S1

Elad Noor[1,†], Hulda S. Haraldsdóttir[2,†], Ron Milo[1,*], Ronan M.T. Fleming[2,*]
1 Plant Sciences Department, Weizmann Institute of Science, Rehovot, Israel
2 Center for Systems Biology, University of Iceland, Reykjavik, Iceland
3 Luxembourg Centre for Systems Biomedicine, University of Luxembourg, Esch-sur-Alzette, Luxembourg
† Both authors contributed equally to this work
∗ E-mail: Corresponding ron.milo@weizmann.ac.il, ronan.mt.fleming@gmail.com

Contents

1 Training data **1**
1.1 Errors associated with the inverse Legendre transform . . . 2
1.2 Weighing the training observations . . . 2

2 Group decomposition **2**

3 Full mathematical derivation of the component contribution method **3**

4 Estimation of error in group model **4**
4.1 Proof that $P_{\mathcal{N}(S)} \cdot P_{\mathcal{N}(\mathcal{G}^\top S)} = P_{\mathcal{N}(S)}$ . . . 5
4.2 Error in current group model . . . 5

5 Reaction type statistics **5**

6 Prediction of flux distributions **7**
6.1 iAF1260 . . . 7
6.2 Recon 1 . . . 7

7 Calculation of confidence and prediction intervals **7**
7.1 Assumptions . . . 8
7.2 Estimation of the distribution of $\Delta_r G^\circ_{obs}$ . . . 8
7.3 Confidence intervals for $\Delta_r G^\circ_x$ . . . 8
7.4 Prediction intervals for $\Delta_r G^\circ_{obs,x}$ . . . 9

8 Symbols **9**

1 Training data

The component contribution method is based on a machine learning algorithm, and completely relies on empirical data in order to infer the parameters needed for later estimation. This empirical data is called the *training data*, and the inference of parameters is called the *training stage*.

Nearly all Gibbs energy measurements, for compounds and reactions in aqueous solutions at near-room temperature, are derived from the equilibrium constants of enzyme-catalyzed reactions. Typically, an enzyme that specifically catalyzes a certain reaction is purified and added to a medium that contains the reaction substrates. After the reaction reaches equilibrium, all reactant concentrations are measured. The equilibrium constant K' is defined as the ratio between the product of all product concentrations and the product of all substrate concentrations. Since there is no easy way to distinguish between pseudoisomers of the same compound, the concentration of every reactant is actually the sum of all its protonation

states. Therefore, K' is the *apparent* equilibrium constant, which is related to the standard *transformed* Gibbs energy of reaction ($\Delta_r G'^\circ$ [1]). The problem with using this data as-is lies in the fact that K' and $\Delta_r G'^\circ$ depend on the aqueous environment (e.g. pH, ionic strength, and pMg). The measurements listed in TECRDB span a wide range of pH and ionic strength values, and many of the reactions have only been measured in non-standard conditions.

In order to standardize the training data and eliminate the effects of pH and ionic strength, we apply an inverse Legendre transform [2] to convert $\Delta_r G'^\circ$ into $\Delta_r G^\circ$ (i.e. from *transformed* Gibbs energies to *chemical* Gibbs energies). As with the forward Legendre transform, this process requires the pK_a values for each of the reactants, provided by Caluclator Plugins from ChemAxon.

The TECRDB contains close to 4000 unique values of K' (for about 400 reactions in different conditions). In addition, the standard chemical Gibbs energies of formation for another ≈ 200 compounds are listed in the literature [3,4]. All this training data is collected and stored in a 1362 by 4146 stoichiometric matrix (S), along with the 4146 Gibbs energies ($\Delta_r G^\circ_{obs}$) corresponding to the columns of S.

1.1 Errors associated with the inverse Legendre transform

It is difficult to verify that effectiveness of the inverse Legendre transform in normalizing the effects of pH and ionic strength. This procedure relies on the reported pH and I that appear in TECRDB, and which are not always correct or even missing altogether. In addition, we ignore the effects of temperature and metal ions in the solution. Perhaps the biggest uncertainty, however, comes from the proton dissociation constants (pK_a values) which we obtain from a 3rd party software tool by ChemAxon.

In theory, if the inverse transform were 100% correct (and all measurements were exact), then multiple measurements of K', for the same reaction at different conditions, would all yield the exact same value for $\Delta_r G^\circ$. To test that, we compared between the standard deviations of the observed Gibbs energies before and after the inverse transform, namely $\sigma(\Delta_r G'^\circ_{obs})$ versus $\sigma(\Delta_r G^\circ_{obs})$. We only looked at reactions with more than 5 repeated measurements. We found that there was a reduction in the median standard deviation from 2.2 kJ/mol to 1.5 kJ/mol (see Figure S1). These results indicate that the condition dependency of K' has been somewhat normalized but that there is still noise in the data, probably attributed both to measurement errors and inaccuracies in the inverse Legendre transform.

1.2 Weighing the training observations

Since we are combining multiple sources of standard Gibbs energies (formation energies, reduction potentials, and inverse-transformed reaction energies from hundreds of publications) we allow our method to factor in how reliable each observation is. Prior to applying the component contribution method, each row of the stoichiometric matrix S is scaled by the confidence of that observation and the corresponding observed values ($\Delta_r G^\circ_{obs}$) are scaled accordingly. The results reported in this paper were derived when applying a fixed weight (e.g. 1) to all observations.

2 Group decomposition

In all group contribution methods, including the component contribution method, molecules are decomposed into non-overlapping structural groups. The contribution of each group to the overall Gibbs energy of the molecule is assumed to be a constant value, independent of molecular environment or the neighboring groups. The implementation by Jankowski et al. [5] added *interaction factors*, which are similar to groups except that they are allowed to overlap with other groups and with each other. In [6], we introduced the notion of pseudoisomeric groups, which are sensitive to the protonation state of the atoms in the group. For the purpose of group decomposition, and the derivation of the group incidence matrix $\mathcal{G}$, we use here the exact same group definitions as in [6].

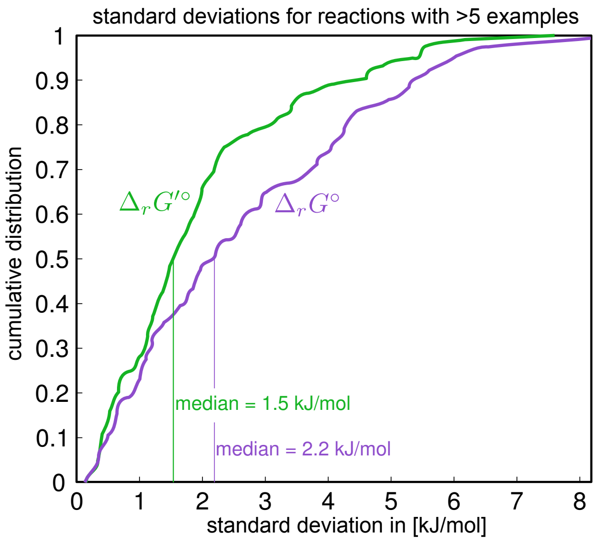

Figure S1. Cumulative distribution functions of the standard deviations for $\Delta_r G^\circ$ and $\Delta_r G'^\circ$. The biochemical Gibbs energies ($\Delta_r G'^\circ$) directly derived from the apparent K' in TECRDB has a higher median standard deviation, 2.2 kJ/mol, than the Gibbs energies after applying the inverse transform ($\Delta_r G^\circ$) – which is 1.5 kJ/mol. Only reactions with more than 5 measurements were considered for this comparison.

3 Full mathematical derivation of the component contribution method

In order to derive the main result for the component contribution method (Eq. 10 in the main text) we reiterate the set of definitions required. Let $S \in \mathbb{R}^{m \times n}$ be the stoichiometric matrix of measured reactions, $\mathcal{G} \in \mathbb{R}^{m \times g}$ be the group incidence matrix, $\Delta_r G^\circ_{obs} \in \mathbb{R}^{n \times 1}$ be the measured standard Gibbs energies of the reactions in S, and $x \in \mathbb{R}^m$ be a stoichiometric vector for a new reaction. Define $P_{\mathcal{R}(S)}$, $P_{\mathcal{N}(S^\top)} \in \mathbb{R}^{m \times m}$ as the orthogonal projection matrices onto the range of S and the null space of $S^\top$, respectively. Define $x_R \equiv P_{\mathcal{R}(S)} \cdot x$ and $x_N \equiv P_{\mathcal{N}(S^\top)} \cdot x$. Equations 3 and 8 (from the main text) state that

$$\Delta_f G^\circ_{rc} = \left(S^\top\right)^+ \cdot \Delta_r G^\circ_{obs},$$

and

$$\Delta_g G^\circ_{gc} = \left(S^\top \mathcal{G}\right)^+ \cdot \Delta_r G^\circ_{obs}.$$

Therefore, the component contribution estimation for the standard Gibbs energy of x will be

$$\begin{aligned}
\Delta_r G^\circ_{cc,x} &= x_R^\top \cdot \Delta_f G^\circ_{rc} + x_N^\top \mathcal{G} \cdot \Delta_g G^\circ_{gc} \\
&= x_R^\top \left(S^\top\right)^+ \cdot \Delta_r G^\circ_{obs} + x_N^\top \mathcal{G} \left(S^\top \mathcal{G}\right)^+ \cdot \Delta_r G^\circ_{obs} \\
&= \left(P_{\mathcal{R}(S)} \cdot x\right)^\top \left(S^\top\right)^+ \cdot \Delta_r G^\circ_{obs} + \left(P_{\mathcal{N}(S^\top)} \cdot x\right)^\top \mathcal{G} \left(S^\top \mathcal{G}\right)^+ \cdot \Delta_r G^\circ_{obs} \\
&= x^\top \left(P_{\mathcal{R}(S)} \left(S^\top\right)^+ + P_{\mathcal{N}(S^\top)} \mathcal{G} \left(S^\top \mathcal{G}\right)^+\right) \cdot \Delta_r G^\circ_{obs}.
\end{aligned} \tag{1}$$

We can thus say the component contribution method is equivalent to a linear regression problem where the contribution coefficients are given by

$$\Delta_c G^\circ_{cc} \equiv \left(P_{\mathcal{R}(S)} \left(S^\top\right)^+ + P_{\mathcal{N}(S^\top)}\mathcal{G}\left(S^\top \mathcal{G}\right)^+\right) \cdot \Delta_r G^\circ_{obs}.$$

4 Estimation of error in group model

The group model in Eq. 7 (main text) relies on the assumption that the standard Gibbs energy of a compound is a simple linear combination of the Gibbs energy contributions of substructures in that compound. Throughout the main text, we have referred to this as the assumption of group additivity. The error resulting from this assumption, which we will refer to as modeling error, can be estimated by decomposing the residual in group contribution fitted Gibbs energies for reactions in S,

$$e_{gc} = \Delta_r G^\circ_{obs} - \Delta_r G^\circ_{gc}, \tag{2}$$

into two components; one corresponding to experimental error and the other to modeling error. The component corresponding to experimental error is exactly the residual in the reactant contribution fitted Gibbs energies for reactions in S,

$$e_{rc} = \Delta_r G^\circ_{obs} - \Delta_r G^\circ_{rc}. \tag{3}$$

An estimate of the modeling error e_m is therefore given by the difference between the two residuals

$$e_m = e_{gc} - e_{rc} = \Delta_r G^\circ_{rc} - \Delta_r G^\circ_{gc}. \tag{4}$$

To clarify we reiterate that $\Delta_r G^\circ_{rc}$, given by

$$\Delta_r G^\circ_{rc} = P_{\mathcal{R}(S^\top)} \cdot \Delta_r G^\circ_{obs}, \tag{5}$$

is the closest point to $\Delta_r G^\circ_{obs}$ that is in the range of $S^\top$ and therefore consistent with the first law of thermodynamics (see main text section *Reactant contribution method*). Since we assume that the first law holds, we must assume that any component of $\Delta_r G^\circ_{obs}$ that is orthogonal to $\mathcal{R}\left(S^\top\right)$ is due to experimental error. The residual e_{rc} is the orthogonal projection of $\Delta_r G^\circ_{obs}$ onto $\mathcal{N}(S)$ which is the orthogonal complement of $\mathcal{R}\left(S^\top\right)$. This can be seen by inserting Eq. 5 into Eq. 3;

$$e_{rc} = \Delta_r G^\circ_{obs} - P_{\mathcal{R}(S^\top)} \cdot \Delta_r G^\circ_{obs} = \left(I - P_{\mathcal{R}(S^\top)}\right) \cdot \Delta_r G^\circ_{obs} = P_{\mathcal{N}(S)} \cdot \Delta_r G^\circ_{obs}. \tag{6}$$

$e_{rc} \perp \mathcal{R}\left(S^\top\right)$ is therefore an estimate of experimental error in $\Delta_r G^\circ_{obs}$.

Group contribution fitted Gibbs energies for reactions in S are given by

$$\Delta_r G^\circ_{gc} = S^\top \mathcal{G}\left(S^\top \mathcal{G}\right)^+ \cdot \Delta_r G^\circ_{obs} = P_{\mathcal{R}(S^\top \mathcal{G})} \cdot \Delta_r G^\circ_{obs}, \tag{7}$$

where $P_{\mathcal{R}(S^\top \mathcal{G})} = S^\top \mathcal{G}\left(S^\top \mathcal{G}\right)^+$ is the orthogonal projector onto the range of $S^\top \mathcal{G}$. The residual of the fit is

$$\begin{aligned} e_{gc} &= \Delta_r G^\circ_{obs} - \Delta_r G^\circ_{gc} = \Delta_r G^\circ_{rc} - \Delta_r G^\circ_{gc} + e_{rc} \\ &= P_{\mathcal{R}(S^\top)} \cdot \Delta_r G^\circ_{obs} - P_{\mathcal{R}(S^\top \mathcal{G})} \cdot \Delta_r G^\circ_{obs} + e_{rc} \\ &= \left(P_{\mathcal{R}(S^\top)} - P_{\mathcal{R}(S^\top \mathcal{G})}\right) \cdot \Delta_r G^\circ_{obs} + e_{rc} = e_m + e_{rc}, \end{aligned} \tag{8}$$

with $e_m = \left(P_{\mathcal{R}(S^\top)} - P_{\mathcal{R}(S^\top \mathcal{G})}\right) \cdot \Delta_r G^\circ_{obs}$. The modeling error e_m is therefore the part of $\Delta_r G^\circ_{obs}$ that is consistent with the first law (i.e., is in $\mathcal{R}\left(S^\top\right)$) but is not explained by the group model (is not in $\mathcal{R}\left(S^\top \mathcal{G}\right)$).

Note that $e_m \perp e_{rc}$ since

$$\begin{aligned} e_m^\top \cdot e_{rc} &= \Delta_r G_{obs}^{\circ\top} \cdot \left(P_{\mathcal{R}(S^\top)} - P_{\mathcal{R}(S^\top \mathcal{G})}\right) \cdot P_{\mathcal{N}(S)} \cdot \Delta_r G_{obs}^\circ \\ &= \Delta_r G_{obs}^{\circ\top} \cdot \left(I - P_{\mathcal{N}(S)} - I + P_{\mathcal{N}(\mathcal{G}^\top S)}\right) \cdot P_{\mathcal{N}(S)} \cdot \Delta_r G_{obs}^\circ \\ &= \Delta_r G_{obs}^{\circ\top} \cdot \left(P_{\mathcal{N}(\mathcal{G}^\top S)} - P_{\mathcal{N}(S)}\right) \cdot P_{\mathcal{N}(S)} \cdot \Delta_r G_{obs}^\circ \\ &= \Delta_r G_{obs}^{\circ\top} \cdot \left(P_{\mathcal{N}(\mathcal{G}^\top S)} \cdot P_{\mathcal{N}(S)} - P_{\mathcal{N}(S)} \cdot P_{\mathcal{N}(S)}\right) \cdot \Delta_r G_{obs}^\circ \\ &= \Delta_r G_{obs}^{\circ\top} \cdot \left(P_{\mathcal{N}(S)} - P_{\mathcal{N}(S)}\right) \cdot \Delta_r G_{obs}^\circ = 0 \end{aligned} \quad (9)$$

(see Section 4.1 for a proof that $P_{\mathcal{N}(S)} \cdot P_{\mathcal{N}(\mathcal{G}^\top S)} = P_{\mathcal{N}(S)}$). Therefore,

$$||e_{gc}||^2 = ||e_{rc}||^2 + ||e_m||^2. \quad (10)$$

It is important to emphasize that the residual e_{rc} is only an estimate of experimental error for several reasons. Systems of biochemical reactions may deviate slightly from the assumptions underlying linear regression, but we assume such deviations are small. More importantly, some error may be introduced by the inverse Legendre transform of the experimental data (see Section 1). Since any such error would contribute equally to e_{gc}, however, this would not affect our estimate of e_m. Even if the inverse transform introduced no error, it is possible that the orthogonal projection of $\Delta_r G_{obs}^\circ$ did not give the true $\Delta_r G^\circ$ for reactions in S. The true $\Delta_r G^\circ$ may be some other point in $\mathcal{R}\left(S^\top\right)$ that is further away from $\Delta_r G_{obs}^\circ$. Our estimate of e_m would then be offset by the same distance. Lastly we note that, although it is unlikely that error due to the assumption of group additivity can be avoided in a linear model such as the group model, it is possible that a different choice of groups would lead to a reduction in e_m.

4.1 Proof that $P_{\mathcal{N}(S)} \cdot P_{\mathcal{N}\left(\mathcal{G}^\top S\right)} = P_{\mathcal{N}(S)}$

$\mathcal{N}(S)$ is a subspace of $\mathcal{N}\left(\mathcal{G}^\top S\right)$, since any $x \in \mathcal{N}(S)$ would have $\mathcal{G}^\top S x = \mathcal{G}^\top 0 = 0$ which would mean that $x \in \mathcal{N}\left(\mathcal{G}^\top S\right)$. Therefore, projecting anything onto $\mathcal{N}\left(\mathcal{G}^\top S\right)$ and then onto $\mathcal{N}(S)$ would be equivalent to projecting it directly onto $\mathcal{N}(S)$.

4.2 Error in current group model

We estimated the root-mean-square modeling error for the group model used in the current publication as

$$\sqrt{\frac{||e_m||^2}{n - \mathrm{rank}(S^\top \mathcal{G})}} = 6.8\,\mathrm{kJ/mol}.$$

The modeling error was approximately normally distributed (Figure S2a) with mean ± stdev = -0.4 ± 6.6. The magnitude of the error was only weakly correlated with the length of group vectors for reactions in S (Pearson's correlation coefficient = 1.9, Figure S2b).

5 Reaction type statistics

The uncertainty in our estimations depends on the quality of the training data (i.e. the measurement error), the amount of examples for each parameter and the crudeness of the assumptions made throughout the evaluation. The hierarchical nature of the component contribution method can be understood by defining four sets of reactions (where S is the stoichiometric matrix for the training data and $\mathcal{G}$ is the group incidence matrix):

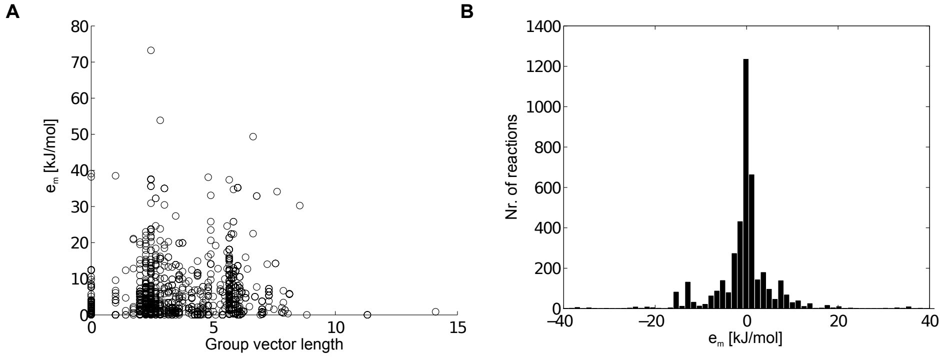

Figure S2. Characteristics of the modeling error e_m for the group model used in the current publication. (A) A scatter plot of e_m against the length of group vectors in $S^\top \mathcal{G}$. (B) A histogram showing the distribution of e_m for all reactions in S.

A: Reaction x is part of the training set (i.e. appears in TECRDB). This is equivalent to saying x is one of the columns in S.

B: Reaction x can be constructed by a linear combination of reactions in the training set (i.e. x is in the range of S). This is equivalent to saying $x \perp \mathcal{N}(S^\top)$.

C: The group decomposition of reaction x can be constructed by a linear combination of reactions decompositions in the training set. This is equivalent to saying $\mathcal{G}^\top x \perp \mathcal{N}(S^\top \mathcal{G})$.

D: x is a reaction.

These four sets have the following layered relationship: A $\subseteq$ B $\subseteq$ C $\subseteq$ D. The first and last relations are trivial. To prove that B $\subseteq$ C, we only need to see that $\forall x \in$ B, $\exists v$ such that $Sv = x$. Then to show that $\mathcal{G}^\top x \perp \mathcal{N}(S^\top \mathcal{G})$, it is enough to see that $\forall y \in \mathcal{N}\left(S^\top \mathcal{G}\right) \Rightarrow \left(\mathcal{G}^\top x\right)^\top y = \left(v^\top S^\top \mathcal{G}\right) y = v^\top \left(S^\top \mathcal{G}\right) y = 0$.

This hierarchy of reactions is ordered by increasing uncertainty levels. Estimates for reactions in A have the lowest uncertainty since these reactions have been directly measured. Estimates for reactions in B have a slightly higher uncertainty, since some deduction is made in order to get the value of $\Delta_r G^\circ$, namely an inverse Legendre transform (which depends on pK_a data) and the projection of the observations onto the column-space of S. CC will apply the RC method exclusively for all reactions in this set. Estimates for reactions in C generally have a higher uncertainty than estimates for reactions in B, as we need to assume that the group decomposition is the only parameter affecting $\Delta_r G^\circ$ – i.e. that each group has a defined $\Delta_{gr} G^\circ$ regardless of its surroundings within the molecule. Note, that every reaction in B is also in C and CC will always use the more precise method wherever possible. Furthermore, if x is in C but not completely orthogonal to $\mathcal{N}(S^\top)$, CC will use GC for the projection of x onto $\mathcal{N}(S^\top)$ and RC for the rest (the part which is orthogonal to this null-space, i.e. contained in B). Reactions that are not in C cannot be evaluated at all, and thus the uncertainty in their $\Delta_r G^\circ$ is ∞. A summary of the four sets of reactions are given in Table S1, along with the fractions of reactions in iAF1260 and Recon 1 that belong to each set.

Table S1. Statistics for the four sets of reactions.

Set	Definition	Methods available	Fraction of reactions iAF1260	Recon 1
A	$\exists i$ s.t. $x = S_{*,i}$	RC / GC / CC	6%	4%
B	$x \perp \mathcal{N}(S^\top)$	RC / GC / CC	9%	8%
C	$\mathcal{G}^\top x \perp \mathcal{N}(S^\top \mathcal{G})$	GC / CC	90%	72%
D	$x \in \mathbb{R}^m$	None	100%	100%

6 Prediction of flux distributions

6.1 iAF1260

Flux distributions in iAF1260 were predicted using loopless flux balance analysis (ll-FBA) [7]. ll-FBA was used in preference to standard FBA [8] to avoid artificially high fluxes through reactions in thermodynamically infeasible loops. Thermodynamics-based metabolic flux analysis (TMFA) [9] was not used to avoid biasing solutions with our estimated Gibbs energies. A total of 312 flux distributions were predicted for iAF1260, corresponding to optimal growth on 174 carbon sources, 78 nitrogen sources, 49 phosphate sources, and 11 sulfur sources that the model was previously shown to grow on [10]. Constraints on exchange, sink and demand reactions in each simulation were the same as in [10]. All simulations were done in Matlab (R2009b, The MathWorks, Natick, MA) with the Gurobi solver (version 5.0.1, Gurobi Optimization, Inc., Houston, TX).

6.2 Recon 1

As growth is not the primary objective of mammalian cells, we did not simulate optimal growth on different nutrient sources for Recon 1. Instead, we predicted optimal flux distributions for 288 metabolic objectives designed to validate Recon 1 [11]. The size of Recon 1 does not allow efficient application of loopless FBA. We therefore applied standard FBA. In an attempt to find loopless flux distributions we searched among all alternative optimal distributions for one with a minimum taxicab norm. As fluxes through reactions in thermodynamically infeasible loops are usually close to the maximum allowed value, flux distributions including such loops are expected to have a greater taxicab norm. Minimizing the taxicab norm will only yield a loopless flux distribution if one exists at the optimum. As a further step to avoid loops, we therefore eliminated all flux distributions where flux through at least one reaction was at the maximum allowed value. This step eliminated all except 97 predicted flux distributions.

7 Calculation of confidence and prediction intervals

In this section we summarize the statistical theory underlying calculation of confidence intervals for the true values of standard reaction Gibbs energies, and prediction intervals for observations of standard reaction Gibbs energies. The summary is based on the textbook by Kutner et al. [12]. We focus the summary on reactant contribution, as it is the simplest linear regression model we work with. Results for group contribution are always analogous to those for reactant contribution. We presented the main results for component contribution in the main text.

7.1 Assumptions

The regression model for reactant contribution is

$$\Delta_r G^\circ_{obs} = S^\top \cdot \Delta_f G^\circ + \varepsilon_{rc}, \tag{11}$$

where $\Delta_r G^\circ_{obs} \in \mathbb{R}^{n\times 1}$ is a vector of observed standard reaction Gibbs energies, $S \in \mathbb{R}^{m\times n}$ is the stoichiometric matrix for reactions in $\Delta_r G^\circ_{obs}$, $\Delta_f G^\circ \in \mathbb{R}^{m\times 1}$ is the vector of standard Gibbs energies of formation for metabolites in S, and $\varepsilon_{rc} \in \mathbb{R}^{n\times 1}$ is a vector of random errors. We assume that the error ε_{rc} is normally distributed, with expected value $E(\varepsilon_{rc}) = 0$, and covariance matrices $\sigma^2(\varepsilon_{rc}) = \sigma^2_{rc} I_n$, where σ^2_{rc} is a constant and I_n is the $n \times n$ identity matrix. Since all element of both S and $\Delta_f G^\circ$ are constants, we have that $\Delta_r G^\circ_{obs}$ is also normally distributed with

$$E(\Delta_r G^\circ_{obs}) = S^\top \cdot \Delta_f G^\circ + E(\varepsilon_{rc}) = S^\top \cdot \Delta_f G^\circ \tag{12}$$

and $\sigma^2(\Delta_r G^\circ_{obs}) = \sigma^2(\epsilon_{rc}) = \sigma^2_{rc} I_n$.

These assumptions about the distributions of errors and observed standard reaction Gibbs energies, apply to any reaction vector $x \in \mathbb{R}^{n\times 1}$, whether it is a column of S or a new reaction. Observed standard Gibbs energies for x are assumed to be distributed as $\Delta_r G^\circ_{obs,x} \sim N\left(x^\top \cdot \Delta_f G^\circ, \sigma^2_{rc}\right)$, and the errors in those observations are assumed to be distributed as $\varepsilon_{rc,x} \sim N\left(0, \sigma^2_{rc}\right)$.

According to the first law of thermodynamics, the true standard Gibbs energies $\Delta_r G^\circ$ of reactions in S are given by

$$\Delta_r G^\circ = S^\top \cdot \Delta_f G^\circ. \tag{13}$$

Comparison with Eq. 11 shows that $E(\Delta_r G^\circ_{obs}) = \Delta_r G^\circ$. The same applies to an arbitrary reaction vector x i.e., that $E\left(\Delta_r G^\circ_{obs,x}\right) = \Delta_r G^\circ_x$. An estimate of $E\left(\Delta_r G^\circ_{obs,x}\right)$ is therefore also an estimate of $\Delta_r G^\circ_x$.

7.2 Estimation of the distribution of $\Delta_r G^\circ_{obs}$

We use the method of least-squares to estimate $E(\Delta_r G^\circ_{obs})$ and σ^2_{rc}. The least-squares fit of the reactant contribution model (Eq. 11) to $\Delta_r G^\circ_{obs}$ gives an estimate of $E(\Delta_r G^\circ_{obs})$:

$$\Delta_r G^\circ_{rc} = S^\top \left(S^\top\right)^+ \cdot \Delta_r G^\circ_{obs}. \tag{14}$$

The variance σ^2_{rc} is estimated as

$$s^2_{rc} = \frac{\|e_{rc}\|^2}{n - rank(S)}, \tag{15}$$

where $e_{rc} = \Delta_r G^\circ_{obs} - \Delta_r G^\circ_{rc}$ is the residual of the fit in Eq. 14.

The reactant contribution estimate of $E\left(\Delta_r G^\circ_{obs,x}\right)$ for an arbitrary reaction vector x is

$$\Delta_r G^\circ_{rc,x} = x^\top \left(S^\top\right)^+ \cdot \Delta_r G^\circ_{obs}. \tag{16}$$

s^2_{rc} from Eq. 15 gives an estimate of the variance in $\Delta_r G^\circ_{obs,x}$, since we assumed that the variance was the same for all x (see Subsection 7.1).

7.3 Confidence intervals for $\Delta_r G^\circ_x$

$\Delta_r G^\circ_{rc,x}$ in Eq. 16 is an estimate of $E\left(\Delta_r G^\circ_{obs,x}\right)$, and thus also of $\Delta_r G^\circ_x$; the true standard Gibbs energy for reaction vector x. However, it is only a point estimate, which is dependent on the particular sample

of observations $\Delta_r G^\circ_{obs}$ in our training set. If we were to repeat the measurements of standard Gibbs energies for all reactions in S, we would get a different estimate of $E\left(\Delta_r G^\circ_{obs,x}\right)$. If we repeated the same measurements an infinite number of times, we could construct the *sampling distribution* of $\Delta_r G^\circ_{rc,x}$.

The sampling distribution of $\Delta_r G^\circ_{rc,x}$ will be normal with mean $E\left(\Delta_r G^\circ_{rc,x}\right) = E\left(\Delta_r G^\circ_{obs,x}\right) = \Delta_r G^\circ_x$, and variance $\sigma^2_{rc,x} = \sigma^2_{rc} \cdot x^\top \left(SS^\top\right)^+ x$ (see [12] for proof). We estimate $E\left(\Delta_r G^\circ_{rc,x}\right)$ as $\Delta_r G^\circ_{rc,x}$, and $\sigma^2_{rc,x}$ as

$$s^2_{rc,x} = s^2_{rc} \cdot x^\top \left(SS^\top\right)^+ x. \tag{17}$$

The estimated standard deviation $s_{rc,x} = \sqrt{s^2_{rc,x}}$ is sometimes referred to as the standard error of $\Delta_r G^\circ_{rc,x}$, and we adopt this terminology here.

The estimated parameters of the sampling distribution of $\Delta_r G^\circ_{rc,x}$ can be used to calculate confidence intervals for $\Delta_r G^\circ_x$. At a specified confidence level $\gamma \in [0\%, 100\%]$, the confidence interval for $\Delta_r G^\circ_x$ is

$$\Delta_r G^\circ_{rc,x} \pm t_{\gamma,\nu} s_{rc,x}, \tag{18}$$

where $t_{\gamma,\nu}$ is the value of a t-distribution with $\nu = n - rank\,(S)$ degrees of freedom, at a cumulative probability of $(100\% + \gamma)/2$. Since ν is large for our reactant contribution model, we can approximate $t_{\gamma,\nu}$ as z_γ; the value of the standard normal distribution at a cumulative probability of $(100\% + \gamma)/2$. The γ confidence interval for $\Delta_r G^\circ_x$ is thus approximated as

$$\Delta_r G^\circ_{rc,x} \pm z_\gamma s_{rc,x}. \tag{19}$$

7.4 Prediction intervals for $\Delta_r G^\circ_{obs,x}$

We seek to validate the reactant contribution method against experimental data. The only experimental data available to us is $\Delta_r G^\circ_{obs}$; which we assume to be a vector of independent observations of standard reaction Gibbs energies. The appropriate way to validate the reactant contribution method is thus to test its ability to predict independent observations of standard reaction Gibbs energies. We assume that independent observations of the standard Gibbs energy for reaction vector x, are normally distributed with mean $E\left(\Delta_r G^\circ_{obs,x}\right)$ and variance σ^2_{rc}. In Subsection 7.2 we estimated $E\left(\Delta_r G^\circ_{obs,x}\right)$ as $\Delta_r G^\circ_{rc,x}$, and σ^2_{rc} as s^2_{rc}. The estimated standard deviation of $\Delta_r G^\circ_{obs,x}$ is $s_{rc} = \sqrt{s^2_{rc}}$.

Based on these estimates, we could mistakenly predict that approximately 68.4% of $\Delta_r G^\circ_{obs,x}$ would fall within the interval $\Delta_r G^\circ_{rc,x} \pm s_{rc}$, approximately 95% would fall within the interval $\Delta_r G^\circ_{rc,x} \pm 1.96 \times s_{rc}$, and so on. In other words, we could assume that the γ prediction interval for $\Delta_r G^\circ_{obs,x}$ were $\Delta_r G^\circ_{rc,x} \pm z_\gamma s_{rc}$. The reason this is incorrect is that $\Delta_r G^\circ_{rc,x}$ is only a point estimate of $E\left(\Delta_r G^\circ_{obs,x}\right)$. Prediction intervals for $\Delta_r G^\circ_{obs,x}$ must account for the variance $\sigma^2_{rc,x}$, of the sampling distribution for $\Delta_r G^\circ_{rc,x}$. The correct way to calculate the γ prediction interval for $\Delta_r G^\circ_{obs,x}$ is therefore as

$$\Delta_r G^\circ_{rc,x} \pm z_\gamma \sqrt{s^2_{rc} + s^2_{rc,x}}. \tag{20}$$

8 Symbols

Table S2. Descriptions of used symbols.

Symbol	Description
R	the gas constant (8.31 J mol^{-1} K^{-1})
T	temperature (in K)
K'	apparent reaction equilibrium constant
Q	reaction quotient
$\Delta_f G^\circ$	standard Gibbs energy of formation (in kJ/mol)
$\Delta_r G^\circ$	standard Gibbs energy of reaction (in kJ/mol)
$\Delta_r G'^\circ$	standard transformed Gibbs energy of reaction (in kJ/mol)
S	the stoichiometric matrix of measured reactions
$\mathcal{G}$	the group incidence matrix
$\Delta_r G^\circ_{obs}$	observed standard Gibbs energy of measured reactions in S
ε_{rc}	deviation of $\Delta_r G^\circ_{obs}$ from the unknown true Gibbs energies
ε_{gc}	deviation of $\Delta_r G^\circ_{obs}$ from the group contribution assumption
e_{rc} / e_{gc}	residual values for the linear model used in RC/GC
$\Delta_f G^\circ_{rc}$	RC estimates of standard Gibbs energies of formation for compounds in S
$\Delta_g G^\circ_{gc}$	standard Gibbs energy contributions of the groups in $\mathcal{G}$
$\Delta_r G^\circ_{rc}$ / $\Delta_r G^\circ_{gc}$ / $\Delta_r G^\circ_{cc}$	RC/GC/CC fitted standard Gibbs energies for reactions in S
$\Delta_r G^\circ_{rc,x}/\Delta_r G^\circ_{gc,x}/\Delta_r G^\circ_{cc,x}$	RC/GC/CC estimation for the standard Gibbs energy of reaction x
$P_{\mathcal{R}(S)}$	orthogonal projection matrix on the range of S
$P_{\mathcal{N}(S^\top)}$	orthogonal projection matrix on the null space of $S^\top$
s^2_{rc} / s^2_{gc}	estimated variance of the error term ε_{rc} / ε_{gc}
V_{rc} / V_{gc}	the covariance matrix for RC/GC estimates
$s_{rc,x}$ / $s_{gc,x}$ / $s_{cc,x}$	the standard error of $\Delta_r G^\circ_{rc,x}$ / $\Delta_r G^\circ_{gc,x}$ / $\Delta_r G^\circ_{cc,x}$

References

1. Alberty RA (2002) Thermodynamics of systems of biochemical reactions. Journal of theoretical biology 215: 491–501.

2. Alberty RA (2002) Inverse Legendre Transform in Biochemical Thermodynamics: Illustrated with the Last Five Reactions of Glycolysis. The Journal of Physical Chemistry B 106: 6594–6599.

3. Thauer RK, Jungermann K, Decker K (1977) Energy conservation in chemotrophic anaerobic bacteria. Bacteriological reviews 41: 809.

4. Alberty RA (2006) Biochemical Thermodynamics: Applications of Mathematica (Methods of Biochemical Analysis). Wiley-Interscience, 480 pp.

5. Jankowski MD, Henry CS, Broadbelt LJ, Hatzimanikatis V (2008) Group contribution method for thermodynamic analysis of complex metabolic networks. Biophysical journal 95: 1487–99.

6. Noor E, Bar-Even A, Flamholz A, Lubling Y, Davidi D, et al. (2012) An integrated open framework for thermodynamics of reactions that combines accuracy and coverage. Bioinformatics (Oxford, England) 28: 2037–2044.

7. Schellenberger J, Lewis NE, Palsson BO (2011) Elimination of thermodynamically infeasible loops in steady-state metabolic models. Biophysical journal 100: 544–53.

8. Varma A, Palsson BO (1994) Metabolic Flux Balancing: Basic Concepts, Scientific and Practical Use. Nature Biotechnology 12: 994–998.

9. Henry CS, Broadbelt LJ, Hatzimanikatis V (2007) Thermodynamics-based metabolic flux analysis. Biophysical journal 92: 1792–805.

10. Feist AM, Henry CS, Reed JL, Krummenacker M, Joyce AR, et al. (2007) A genome-scale metabolic reconstruction for Escherichia coli K-12 MG1655 that accounts for 1260 ORFs and thermodynamic information. Molecular systems biology 3: 121.

11. Duarte NC, Becker SA, Jamshidi N, Thiele I, Mo ML, et al. (2007) Global reconstruction of the human metabolic network based on genomic and bibliomic data. Proceedings of the National Academy of Sciences of the United States of America 104: 1777–82.

12. Kutner MH, Nachtsheim CJ, Neter J, Li W (2004) Multiple Regression I. In: Applied Linear Statistical Models, McGraw-Hill/Irwin, chapter 6. 5 edition, pp. 214–255.
